# Ferroelectric-Paraelectric Transition In A Membrane With Quenched-Induced δ-Phase Of PVDF

**DOI:** 10.1038/s41598-017-06044-y

**Published:** 2017-07-17

**Authors:** O. García-Zaldívar, T. Escamilla-Díaz, M. Ramírez-Cardona, M. A. Hernández-Landaverde, R. Ramírez-Bon, J. M. Yañez-Limón, F. Calderón-Piñar

**Affiliations:** 1 Centro de Investigación y de Estudios Avanzados del I.P.N., Unidad Querétaro. Libramiento Norponiente No. 2000, Fracc. Real de Juriquilla, Querétaro, Qro., C.P., 76230 Mexico; 2 Facultad de Física/IMRE, Universidad de La Habana, San Lázaro y L, La Habana, C.P. 10400 Cuba; 3 Centro de Investigaciones en Ciencias de la Tierra y Materiales, Universidad Autónoma del Estado de Hidalgo (UAEH), Ciudad del Conocimiento, Col. Carboneras, 42184 Mineral de la Reforma, Hgo. Mexico

## Abstract

The stabilization of δ-phase of poly(vinylidene fluoride) PVDF in a 14 µm-thickness ferroelectric membrane is achieved by a simple route based on the use of a dimethylformamide (DMF)/acetone solvent, in which the application of external electric field is not required. X-ray diffraction and calorimetric experiments on heating reveal that, at 154 °C, the original mixture between ferroelectric δ-phase and paraelectric α-phase transits to a system with only this latter phase in the crystalline fraction. A gradual and slight increment of amorphous fraction up to the melting at 161 °C is also observed. The existence of δ-phase is corroborated by the occurrence of a broad maximum around 154 °C in dielectric permittivity measurements, as well as the hysteresis loops observed at room temperature. These results suggest a wide thermal window for a stable δ-phase, between room temperature and 154 °C, a subsequent transition into α-phase and the corresponding melting at 161 °C. The broad dielectric maximum observed around 154 °C in dielectric and calorimetric measurements, can be associated with a diffuse ferroelectric-paraelectric transition.

## Introduction

In recent years, ferroelectric polymers have been of great interest due to its their promising potential applications in advanced technologies such as transducers, actuators, memory devices, sensors and high energy density capacitors^1, 2^. In general, compared with conventional ceramics, ferroelectric polymers (such as polyamides (odd nylons), cyanopolymers, polyureas, polythioureas and Poly (vinylidene fluoride) (PVDF) and its copolymer trifluoroethylene (TrFE)^3^) show some advantages for specific applications that are worth mentioning: they are lightweight materials, flexible, moldable, chemically stable and resistant, possess low acoustic impedance. Moreover, their synthesis has been successfully proved in large-scale manufacture and their structural modifications are relatively easy to achieve. Among them, the Poly(vinylidene fluoride) (PVDF) and its copolymer trifluoroethylene (TrFE) are the most representative. However, their performance on electroactive properties (i.e. pyro, piezo and ferroelectricity) remain below their ceramic counterparts.

Poly(vinylidene fluoride), PVDF (-CH_2_-CF_2_-)_n_, is a semicrystalline polymer, whose ferroelectric nature depends of the dipoles formed between the C-H and C-F bonds, the polymeric chain conformation and the molecular packing of the crystalline region. There are three polymorphs, α, β, and γ, which have been studied extensively and another phase, less studied, called δ^3, 4^. Except α-phase, all others exhibit ferroelectric order^3, 5^.

The most common and easy to obtain is the non-ferroelectric α-phase, which can be obtained directly during crystallization from the melt or during polymerization^3, 6^. It is characterized by a *trans-gauche-trans-gauche*′ (TGTG′) conformation of macromolecular chains with the dipole moments aligned in antiparallel way. The ferroelectric β-phase, which is the most extensively used for piezoelectric and pyroelectric applications, is characterized by an all-*trans* (TTTT) conformation. This phase is usually stabilized and isolated either by mechanical deformation of the α-phase under electric field^7^ or by copolymerization with a small quantity of TrFE or tetrafluoroethylene (TFE)^8, 9^.

The γ-phase is characterized by a sequence of *trans* and *gauche* conformation (TTTGTTTG′) leading to a non-cero net polarization of the unit cell. This phase stabilizes by crystallization from solution in different solvents (with high dipolar moments) or after thermal annealing of α-phase^6^. The γ-phase is difficult to obtain isolated, usually coexists with α-phase and its electroactive properties are lower than those of the β-phase.

The δ-phase is characterized by the same TGTG′ conformation of α, but with a parallel chain dipoles alignment. The difference in the polymer chain packing, respect to α, confers to δ-phase its ferroelectric character^3, 4, 10, 11^ and imply only small changes in the intensities of some diffraction planes and some FTIR absorption bands between them^10^. This phase has been poorly studied and it is stabilized by applying high electric field, for short periods of time, to α-phase^4, 10, 11^.

The research on this ferroelectric phase has not been plentiful due to the technological difficulties for its stabilization. Here, for the first time to our knowledge, we report the δ-phase stabilization without the application of an electric field, as well as a detailed study of the ferroelectric–paraelectric phase transition in δ-phase, through dielectric measurements with temperature.

## Experimental procedure

For the preparation of the membranes, PVDF powder (Sigma – Aldrich Mw~534000) was dissolved in a solution of dimethylformamide (DMF) and acetone (both J. T. Baker), with a volume ratio of 1:9, to obtain a concentration of 25 g/L. The resulting solution was stirred with magnetic bars at room temperature for 2 h. Then, it was stirred in an ultrasonic bath for 8 minutes. Subsequently, the solution was magnetically stirred again at 90 °C until it turned transparent (after 10 min under stirring) and later was cooled keeping the stirring. The transparent solution was poured into a Petri dish with an internal diameter of 6 cm, approximately, and then the mounting was placed over a hot plate at 50 °C during 10 minutes in order to evaporate the solvent. Finally, the sample was heated in a furnace at 200 °C for 1 h, and subsequently quenched (the sample was removed from the furnace and placed in a Petri dish at room temperature). The membrane, transparent and with 14 μm of thickness, was removed from the Petri dish. Specifically, in samples for electrical characterization, gold electrodes were deposited by sputtering on both sides of the membrane.

Phase identification was accomplished by patterns obtained from variable temperature Glancing X-ray Diffraction (GIXRD) experiments conducted on a RIGAKU Ultima IV diffractometer using CuK_α1,2_ radiation (1.5406 Å/1.5444 Å doublet wavelength generated by an X-ray tube operating at 40 kV and 30 mA), in a 2θ range from 10° to 50°, a step scan of 0.02° and an integration time of 0.5 s per step. The parallel incident beam was fixed at an angle of 3° with the sample surface and irradiated area of *ca*. 1 cm^2^. The control of the temperature was set from a high-temperature chamber HT-1500 from RIGAKU, operating within a temperature range between 30 °C and 170 °C, witch a heating rate of 10 °C/min. The membrane was fixed to the flat sample holder with a piece of Kapton polyimide film.

The Raman scattering spectra were recorded at room temperature using a HORIBA xplora plus micro-Raman spectrometer. The measurements were performed with a laser excitation line of 532 nm. FTIR Spectrum was recorded using a GX Perkin Elmer spectrophotometer coupled to autoIMAGE microscope and using an Attenuated Total Reflectance (ATR) technique. Differential Scanning Calorimetry (DSC) was carried out in a Mettler Toledo DSC822e calorimeter in the temperature range from 30 °C to 200 °C in air, using a heating rate of 10 °C/min. A portion of the PVDF membrane was put into a hermetically sealed aluminum crucible with a capacity of 40 μL.

The electric impedance and phase measurements were performed in a Precision Impedance Analyzer Agilent 4294A, in a temperature range from 30 °C to 170 °C. Ferroelectric characterization was performed in a Radiant precision LC coupled to a voltage amplifier TRek 609E-6 at frequency of 20 Hz and different applied fields. Piezoelectric displacement measurements were recorded by MTI 2100 Fotonic Sensor system (with a sensitivity of 2.5 Å) coupled to the Radiant precision LC using the converse piezoelectric effect (i.e., generation of a mechanical displacement under the application of an electric field).

## Results and Discussion

**Figure 1:**
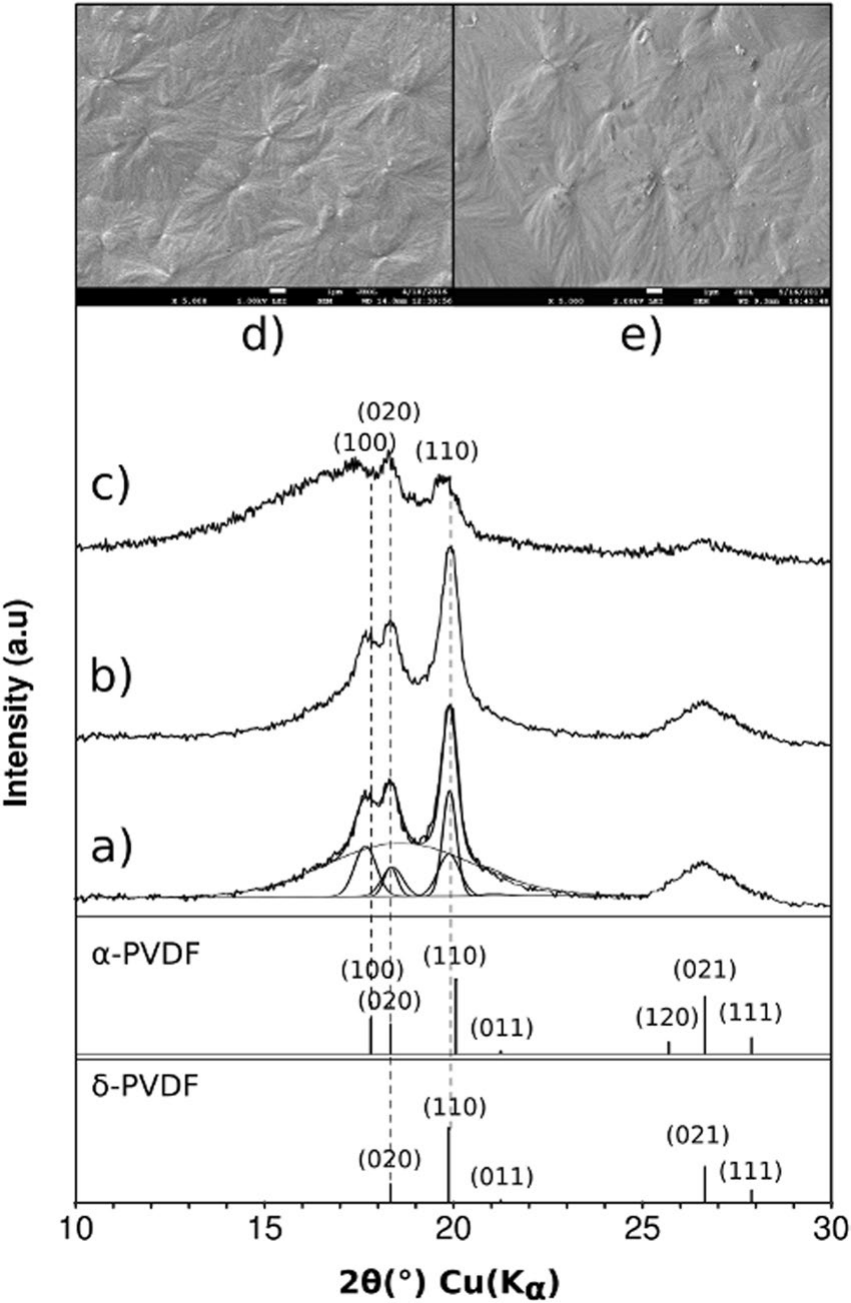
Room temperature X-Ray diffraction patterns of the poly(vinylidene fluoride) membrane indexed as α-phase and δ-phase: (**a**) before and (**b**) after hysteresis measurement; (**c**) at 170 °C. SEM micrographs (**d**) before and (**e**) after hysteresis measurement.

Figure 1 shows the X-ray diffraction patterns (GIXRD) of the membrane before and after hysteresis cycling. Both patterns clearly exhibit the semicrystalline nature of the sample in view of the broad “peak” of the amorphous fraction within the range between 15 and 22° 2θ. Crystalline planes at 2θ = 17.65°, 18.31°, 19.90° and 26.59° are indexed as (100), (020), (110) and (021), respectively, that it is consistent with α-phase (PDF#00-061-1403–ICDD) reported elsewhere^6, 7, 10^ or also named Form II of PVDF in ref. 3, 12, as well as with δ-phase structure. Li *et al*.^10^ reported that both phases are nearly identical, with the unique difference in the space group: centrosymmetric P2_1_/c (space group number 14) and non-centrosymmetric P2_1_cn (space group number 33) for α and δ phases, respectively.

Deconvolution of crystalline peaks, by using Jade-MDI 9.7.0 software, in the range 13–24° 2θ of the GIXD pattern was conducted considering crystal structures of α and δ phases as they are reported in Li *et al*.^10^ (Fig. 1a). FWHM’s of 0.64, 0.42 and 4.57 were estimated for α, δ and amorphous peak, respectively, with unit-cell parameters *a* = 5.0155(7) Ǻ, *b* = 9.6620(0) Ǻ, *c* = 4.6009(16) Ǻ and *β* = 90° for both crystalline phases. These parameters are slightly higher than those reported by Li *et al*.^10^, where they were presented as mutually equal values in both phases. But differences in relative intensities leads to consider a likely coexistence between α and δ phases: intensities of (020) and (110) deconvoluted peaks are, in fact, a result of the influence of both phases.

The diffraction pattern of the film after hysteresis cycling (Fig. 1b) is like that from pristine membrane (Fig. 1a). Thus, in the present work we have not ruled out the concurrent presence of both phases before and after the annealing. In Fig. 1c, pattern of sample at 170 °C clearly shows a remnant of α-phase, as well as a notable increment of amorphous fraction. The crystal growth of α-phase and δ-phase into spherulites can be observed in the SEM micrographs of Fig. 1d and e, for non-polarized and polarized membranes, respectively. These images are microstructural evidences at grain scale of the occurrence of α-phase and, eventually, δ-phase of PVDF^9^.

By comparing, the diffraction pattern of the γ-phase has been characterized by a broad main reflection peak centered around 20° 2θ, with a broad shoulder to the left, centered around 18.5°^12^, while the β-phase is characterized by a single relatively narrow main reflection peak centered around 21°^7, 12^. Based on the results, γ and β phases could be discarded, however, to confirm the previous statement, measurements of Raman and FTIR spectroscopy were performed.

**Figure 2:**
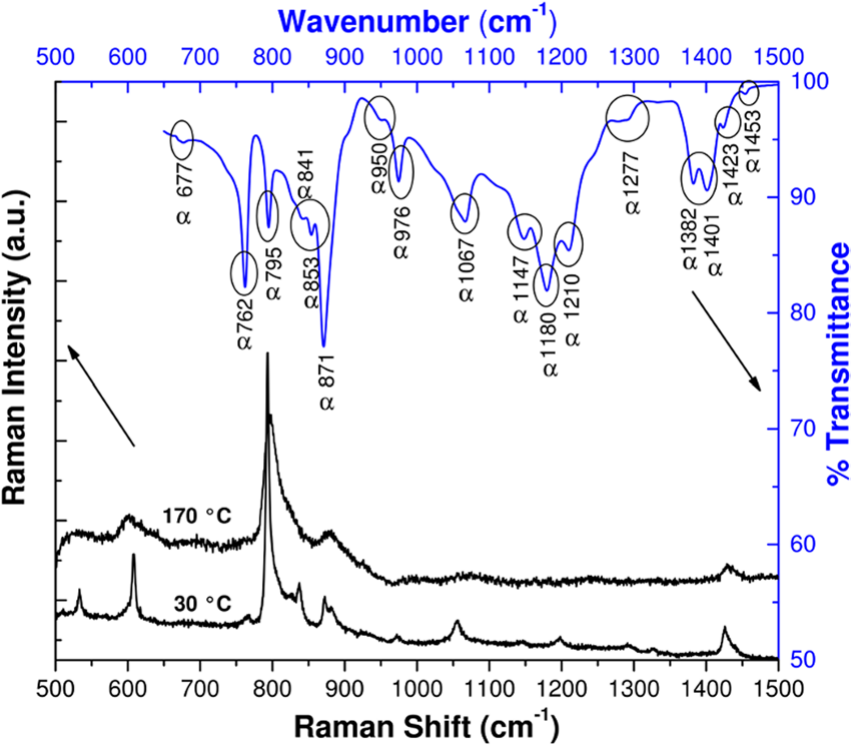
Raman spectra at 30 °C and 170 °C; FTIR spectrum of the membrane at 30 °C.

The Raman and FTIR spectra of the membrane, in the 500–1500 cm^−1^ region, are shown in Fig. 2. No FTIR and Raman experiments were performed on the polarized samples because they would imply oil-soaked membranes and the consequent appearance of bands would interfere with the proper PVDF-phases signals. In the FTIR spectrum of the non-polarized sample, at 30 °C, well-defined absorption bands at 1423, 1401, 1382, 1210, 1180, 1067, 976, 871, 795 and 762 cm^−1^, related to α-phase, are observed^7, 9, 10, 12^. In the graph, other less pronounced bands are ascribed as α-phase crystallization as well. There are neither predominant absorption bands at 812, 882 and 1234 cm^−1^, related to γ crystallization^7, 12^, nor in 840 and 1280 cm^−1^, related to β-phase^7, 9, 12^. The Raman spectrum at 30 °C (Fig. 2) is composed by a family of narrow peaks attributed to δ-phase, which they are combined with broad bands of α-phase^13–15^. This is an apparent spectrum of α-phase, or a mixture of both α and δ phases, as Li *et al*.^10^ stated. At 170 °C, only the broad peaks of α-phase are noticeable.

It is known that these two phases (i.e. α and δ) have similar FTIR spectra and x-ray diffraction (GIXRD) pattern^3, 10, 11^. The subtle differences lie not in the peak positions but in the relative intensities of some absorption bands and crystalline planes^3, 10^. It is therefore difficult to unambiguously differentiate these phases using both above-mentioned techniques. In fact, experimentally, Raman spectroscopy and the ferroelectric character of the sample are the unique evidences in this study to confirm the obtaining of the δ-phase^10, 11^.

**Figure 3:**
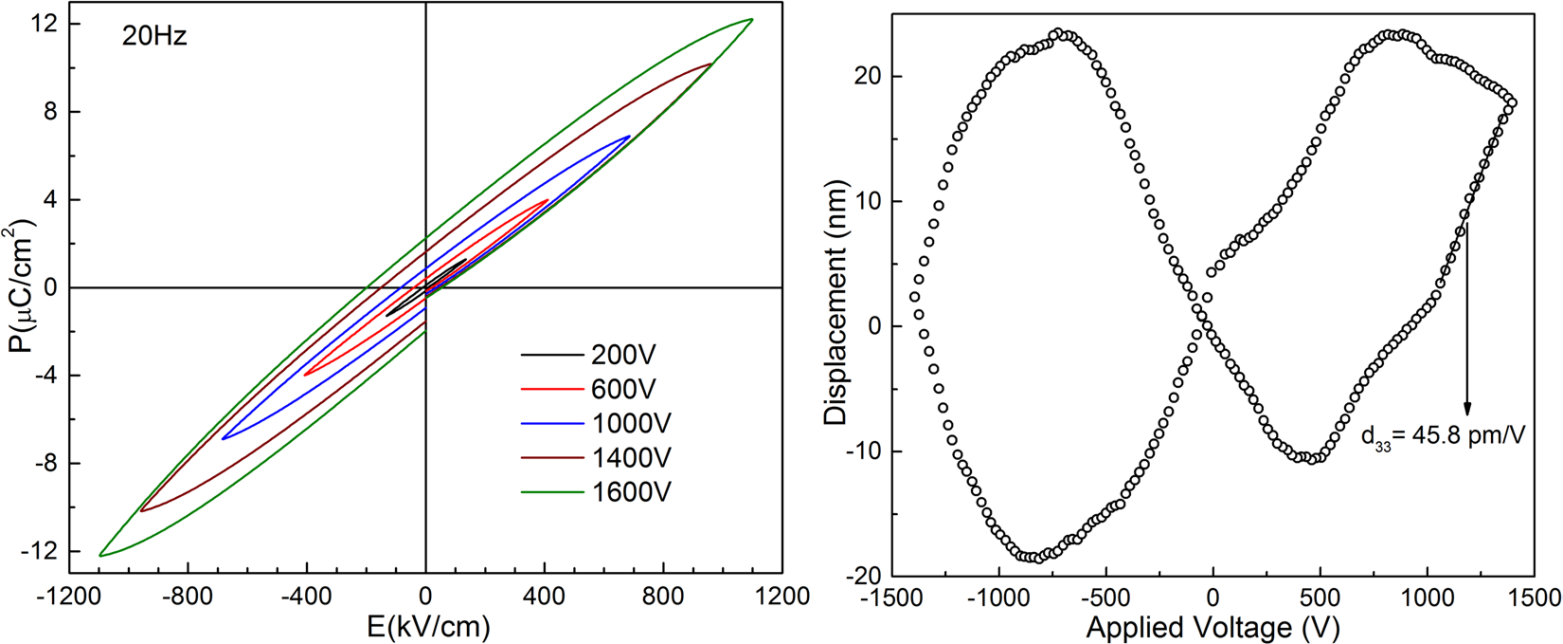
(**a**) Hysteresis loops of the membrane at several applied fields; (**b**) Displacement *vs*. applied voltage.

Figure 3 shows the ferroelectric hysteresis loops, of the membrane, for different applied fields at a frequency of 20 Hz and at room temperature. Surprisingly, the beginning of the hysteresis is located at low bias and the remnant polarization increase gradually with the bias. No paraelectric behavior was observed even at low fields. This result suggests that the pristine membrane has ferroelectric order. Considering Raman and GIXRD results presented above, the ferroelectric behavior is only plausible in a system with δ-phase or a mixture between α and δ phases.

The stabilization of δ-phase by applying high electric fields (i.e. over 1700 kV/cm) to α-phase has been reported elsewhere^4, 10^. During our hysteresis cycling experiments, those high values of fields mentioned above were not reached but the plotting of results in Fig. 3a shows how the inner loops at low values fields are contained in outer loops corresponding to higher fields, an unexpected evidence of the likely formation of δ-phase^4, 10^ during the synthesis process. However, in order to discard the electro-conformation of any polar phase, further X-ray diffraction (GIXRD) measurements were performed to the membrane after the hysteresis cycling (see Fig. 1b).

As can be observed there are not differences between the diffraction patterns before (Fig. 1a) and after the maximum field applied (Fig. 1b). Therefore, it can be ensured that the pristine membrane crystallizes into δ-phase, or a mixture between α and δ phases. This work represents the first report about the obtaining of δ-phase from a direct synthesis, without the application of an electric field. It has been reported elsewhere^3, 6^ that the non-ferroelectric α-phase can be obtained directly during crystallization from the melt, hence, the obtaining of the δ-phase could be a direct consequence of the quenching process used in our synthesis procedure.

In this process, PVDF is heated up to 200 °C and kept at this temperature by one hour, at the end of which the polymer transforms into a viscous liquid. This temperature is high enough to achieve a liquid which, when subjected to a quenching process, “freezes” the δ-phase, which crystallizes in a system of greater symmetry than that of the α-phase. Both phases (i.e. α and δ) present the same molecular conformational sequence TGTG’, but a different arrangement of chains results in differences on crystal symmetry: α-phase crystallizes in the monoclinic group P2_1_/c and δ-phase occurs within the orthorhombic group P2_1_cn; it seems reasonable to assume that the δ-phase derives from a more isotropic liquid. Consequently, experiments from a liquid quenched from a lower temperature than 200 °C would result in a system with a greater fraction of α or this latter one as the unique phase in the system as well.

Additionally, it cannot be ruled out that, during the quenching process of this viscous liquid from 200 °C to room temperature, PVDF solidifies rapidly and adheres tightly to the bottom and walls of the container. The sample shrinks due to the loss of volatiles. Thus, after adhesion, the polymer chains of PVDF are subject to stress and the membrane reduces its thickness but not the diameter. At the end of the quenching process, due to the different thermal expansion coefficients of both the polymer and the glass container, the PVDF membrane probably acquires a residual stress, which partially relaxes after detachment. Therefore, the stress of the PVDF membrane during the quenching process produces a partial transition to the δ-phase. It has been reported that the PVFD α-phase can be transformed to the β one by applying stress of the order of 100–350 MPa^16^. In our case it is difficult to estimate the order of the stress in the quenching process, however, since we did not obtain the β-phase, we can assure the stress is moderate, lower than 100 MPa.

As far as we know, the first report on hysteresis loops of δ-phase of PVDF was made by Li *et al*.^10^ in thin films of about 0.4 µm. They report remnant polarizations P_r_ = 7 µC/cm^2^ and maximum polarizations P_max_ around 12 µC/cm^2^ at maximum electric field of 4000 kV/cm. Our membrane has values of P_max_ = 12.2 µC/cm^2^ and remnant polarization P_r_ = 2.3 µC/cm^2^ at a maximum applied field of 1200 kV/cm. The remnant polarization of the membrane is significantly below from that reported previously^5, 10^, maybe due to the coexistence of α and δ phases, the lower applied maximum field compared to those of references^5, 10^ or both. Thus, the applied field of 1200 kV/cm could be sufficient to reorient the individual dipole moments of the cell of α-phase, but not enough to stabilize the parallel configuration, as the α cell recover its non-ferroelectric antiparallel configuration when the field vanishes to zero. In view of the fact that this maximum field is very close to the coercive field (i.e. Ec = 1150 kV/cm, Li *et al*.^10, 11^ in δ-phase, we infer that dipoles of δ-phase were not completely reoriented.

Furthermore, contrary to other reported δ-PVDF, we do not polarize the sample to obtain the δ-phase. So, in our membrane there is not a preferential orientation of the polar **a** axis in the direction normal to the surface. This fact could be another reason why we do not obtain a square loop with well-defined saturation polarization as reported by^10^.

The displacement versus applied voltage on piezoelectric measurements is show in Fig. 3b. The values of displacements and that of the piezoelectric coefficient d_33_ (determined by the linear slope in Fig. 3b) are similar or higher of that reported for other PVDF-based systems^17, 18^. Even when the coercive field could not be reached, a butterfly-like behavior, typical of polarization switching present in ferroelectric systems, is observed. This result, in completely agreement with the hysteresis loops, suggests that at least a fraction of polar domains can be switched with values of field below the coercive one reported by^10, 11^ and confirm the ferroelectric nature of the membrane.

**Figure 4:**
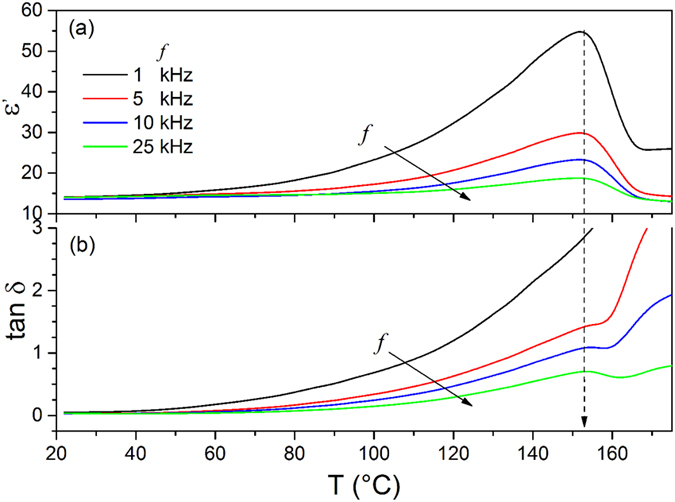
Dielectric measurements of the PVDF membrane at different frequencies.

**Figure 5:**
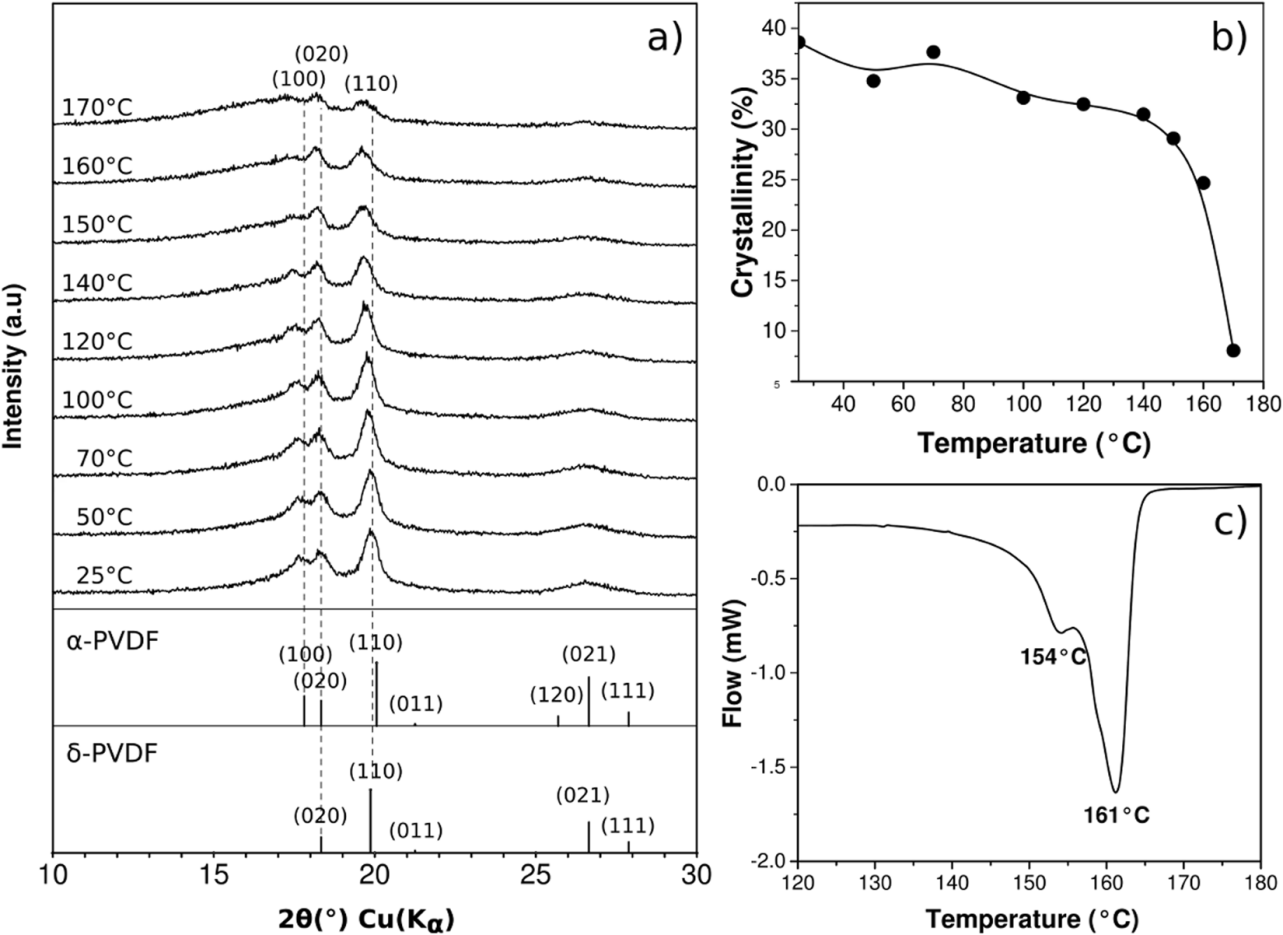
(**a**) GIXRD patterns of PVDF membrane at different temperatures, (**b**) crystallinity *vs*. temperature plot and (**c**) the differential scanning calorimetry (DSC) curve for the PVDF sample.

On the other hand, in order to study the ferroelectric – paraelectric phase transition and the thermal stability of the membrane, besides differential scanning calorimetry (DSC) analysis, additional dielectric and loss permittivity measurements, and Glancing Incidence X-ray diffraction (GIXRD), were conducted with variation of temperature (Figs 4 and 5). Dielectric measurements in a thermal range from room temperature to 170 °C, using several frequencies between 1 kHz and 25 kHz, are shown in Fig. 4. As a remarkably consistent fact, at room temperature, the general plot of Fig. 4 shows values of dielectric permittivity (ε′) of 14 and dielectric losses (tanδ) of 0.03, similar to those reported in ref. 19 for the α, β and γ phases of PVDF.

The dielectric permittivity at 1, 5, 10 and 25 kHz, analyzed as a function of temperature unveils a broad maximum (i. e., ε_max_) around 154 °C (Fig. 4). The maximum value ε_max_ is highly dependent of the probing frequencies but the temperature at which the maximum takes place (T_c_) is frequency independent. Similarly, dielectric losses show a singularity at same temperature than that of ε′, more marked at higher frequencies. At low frequencies, the maximum is masked due to the contribution of dc conductivity to tan δ, but it is clearly observed with increasing frequency. This is supported by the conductive behavior observed in dielectric losses: contrary to dipole behavior, the dielectric losses decrease with increasing frequency. The lack of frequency dependence of T_c_ and the fact that the maximums take places at the same temperature for ε′ and tanδ indicate the occurrence of a ferroelectric-paraelectric phase transition rather than any relaxation process that occurs in the polymer. It is worth to note that earlier studies exhibited that the relaxation process associated to dipolar motion in both the amorphous fraction (α_a_) and that of dipolar motion in the crystalline fraction (α_c_) shift to higher temperature with increasing frequencies^19^. The broad maximum suggests the occurrence of a diffuse ferroelectric – paraelectric phase transition.

Figure 5a shows diffraction patterns measured at different temperatures from room temperature up to 170 °C. The increment of the amorphous fraction with temperature is evident from the broadening of the “glass hump” located roughly between 10 and 25° 2θ. In this sense, an estimation of crystallinity (%) is performed by obtaining the ratio between the area associated to crystalline peaks and the total area under the pattern curve above the background by using Jade-MDI 9.7.0 software^20^. A graphical plot of the crystallinity (%) as function of temperature is presented in the Fig. 5b. It is noteworthy in this graph that the progressive and slight decrease of crystallinity observed at low temperatures is followed by an abrupt change of slope from 140 °C.

The Fig. 5c shows a DSC curve of the membrane. In this thermogram, the onset temperature of the principal complex signal is 140 °C and finishes at 170 °C, including two well defined peaks at 154 °C and 161 °C. The former roughly coincides with the T_c_ observed in dielectrics measurements and the second could be related to melting temperature (T_m_) observed in PVDF-based materials^3^. So, it can be concluded, undoubtedly, that δ-PVDF has a ferroelectric-paraelectric phase transition around 154 °C. These results suggest a wide thermal window for a stable δ-phase, between room temperature and 154 °C.

At 170 °C (i.e., the final part of the melting signal in the DSC curve, the T_f_), the crystallinity (%) results in a small value of only 5%. Differences in sample weights –i.e. 2.17 mg and 32.00 mg for both DSC and GIXRD samples, respectively– and in shapes of heat conductive sample holders – i.e. a capsule in DSC against the flat holder in GIXRD experiments–, as well as the occurrence of a probable annealing effect in membrane through the isothermal GIXRD experiments, are interpreted as the principal causes of the presence of a crystalline remnant detected in the GIXRD experiment at 170 °C. This discrepancy produced by differences in the kinetics of melting is usually encountered in studies which both DSC and GIXRD techniques are combined; in these cases, thermal properties are calculated from DSC experiments and GIXRD appears to be a more convenient method for the determination of crystallinity (%)^21, 22^.

According with the identification of α-phase and δ-phase below the transition at 154 °C and the occurrence of the isolated α-phase above this temperature in crystalline fraction (see deconvolutions of crystalline peaks in Fig. 1a), we visualize the ferroelectric-paraelectric transition as a transformation of δ-phase into α-phase. This latter is transformed, eventually, into amorphous during the melting process. Crystallinity values show a non-lineal evolution on temperature, depending on the phase that it is preponderant within crystalline fraction: crystallinity diminishes faster with the presence of δ-phase. The small thermal window where only α-phase is stable corresponds to the thermal range of paraelectricity (i.e. 154–161 °C), below the melting process (i.e. 161–170 °C, approximately).

## Conclusions

The dielectric, calorimetric, structural and ferroelectric properties of a PVDF membrane, prepared by solution casting method, were investigated. We report the stabilization of the polar δ-phase without the application of an electric field and the study of its ferroelectric phase transition through dielectric, structural and calorimetric measurements with temperature. This report provides a simple and low cost way to access to the stable ferroelectric δ-phase.

GIXRD patterns and FTIR spectroscopy studies discard the presence of the more commons γ and β ferroelectric phases. The membrane stabilizes into a mixture between α and δ phases. The occurrence of these phases was confirmed taking into account the results of GIXRD patterns, DSC, dielectric, FTIR, Raman and hysteresis measurements.

The hysteresis loops observed at room temperature confirms the presence of a ferroelectric phase in the membrane. The remnant polarization is below the reported by Li *et al*., due to the coexistence of α and δ phases, the low fields applied to the membrane and the lack of preferential orientation along the polar **a** axis in the direction normal to the surface. Calorimetric results reveals two well defined peaks at 154 °C and 161 °C. The former coincides with the broad maximum around 154 °C observed in dielectric permittivity and dielectric losses, so it can be related to ferroelectric – paraelectric phase transition temperature of δ-phase. The later peak at 161 °C can be related to the melting process. The results suggest that the ferroelectric δ-phase transforms, at 154 °C, into crystalline α-phase, which melts at 161 °C.
